# Supplementary material for: Characterization of the Gut Microbiota of Individuals at Different T2D Stages Reveals a Complex Relationship with the Host
Source: Microorganisms. 2020 Jan 10;8(1):94. doi: 10.3390/microorganisms8010094 (PMC7022408; doi:10.3390/microorganisms8010094)
Supplement: Supplementary file 1 [file microorganisms-08-00094-s001.pdf]

microorganisms

MDPI

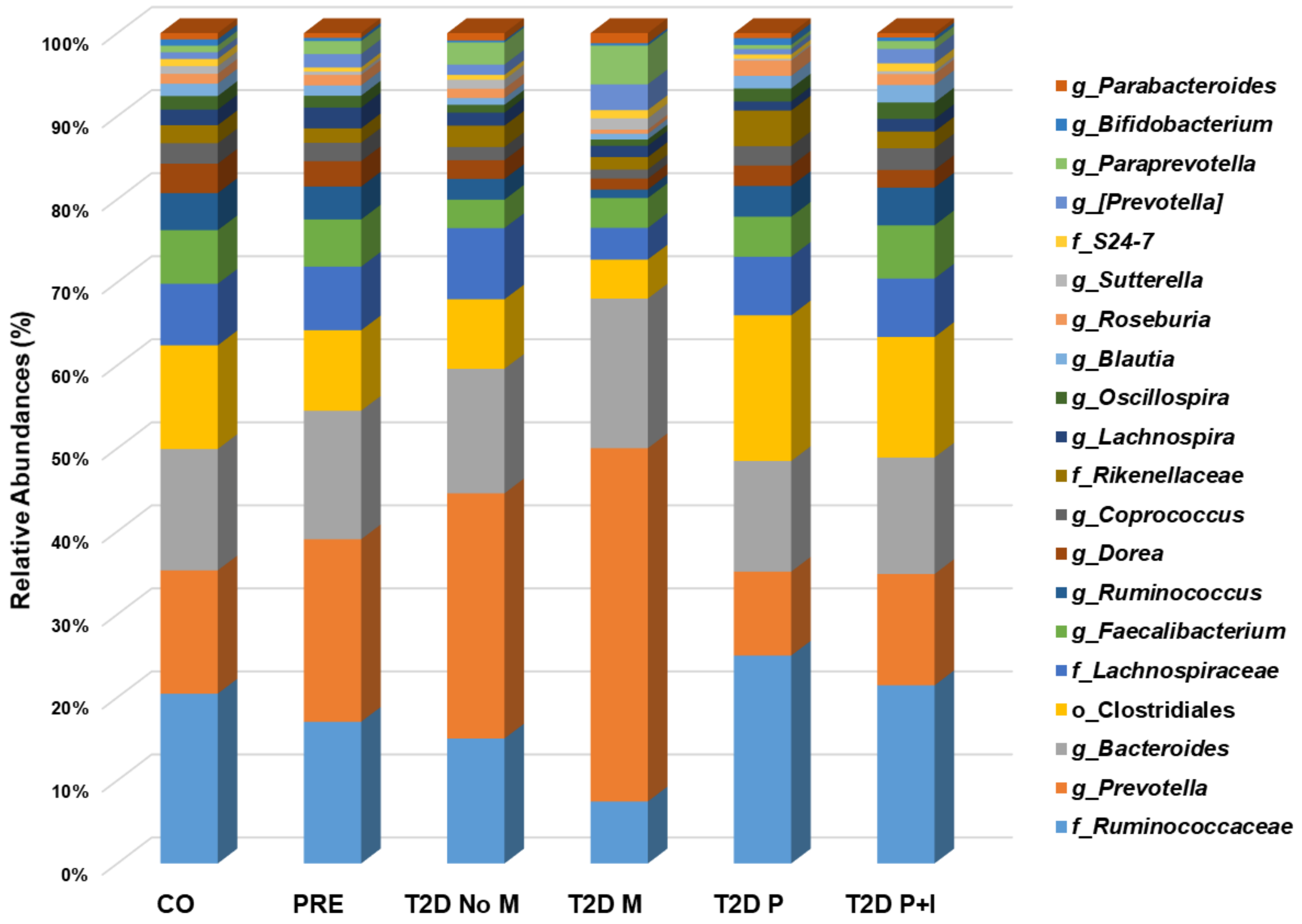

Figure S1. Relative abundances from gut microbiota in the six study groups: CO, control, PRE, prediabetes, T2D−No−M, T2D no medication, T2D−M, T2D−Metformin, T2D−P, T2D polypharmacy, and T2D−P+I, T2D polypharmacy plus insulin. The figure shows bar charts of relative abundances for relevant bacterial orders, families and genera, present in the six phenotypic categories. Each bacterial taxon is identified by color as indicated beside the bars.

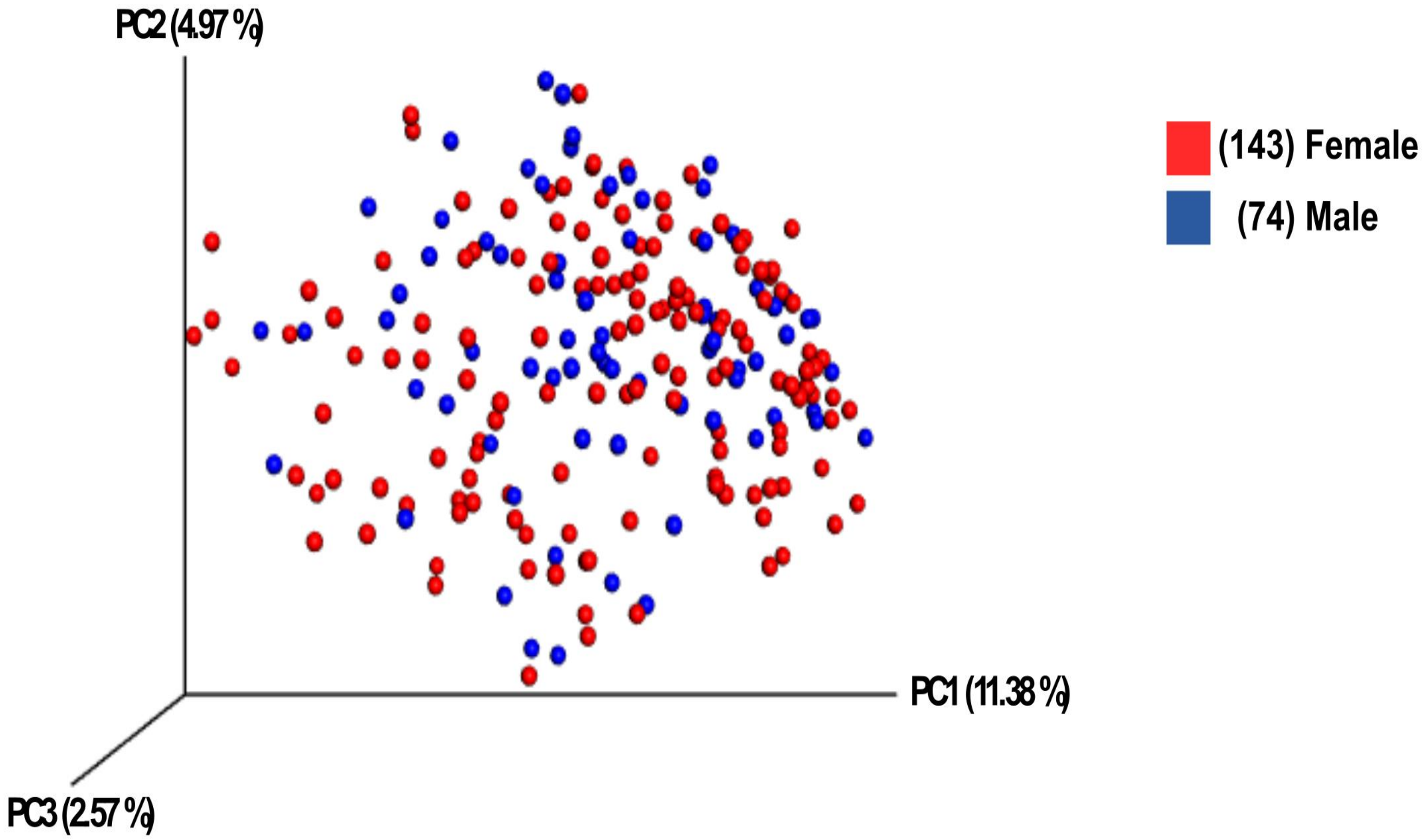

Figure S2 Bacterial beta diversity based on sex. The figure shows a three-dimensional scatter plot, generated using Unweighted UniFrac analyses, showing for each dot, the distance along each of the three principal coordinates (PCoA) axis. Each dot represents the microbial community for each individual of all groups stratified by sex, Red, females, Blue, males.

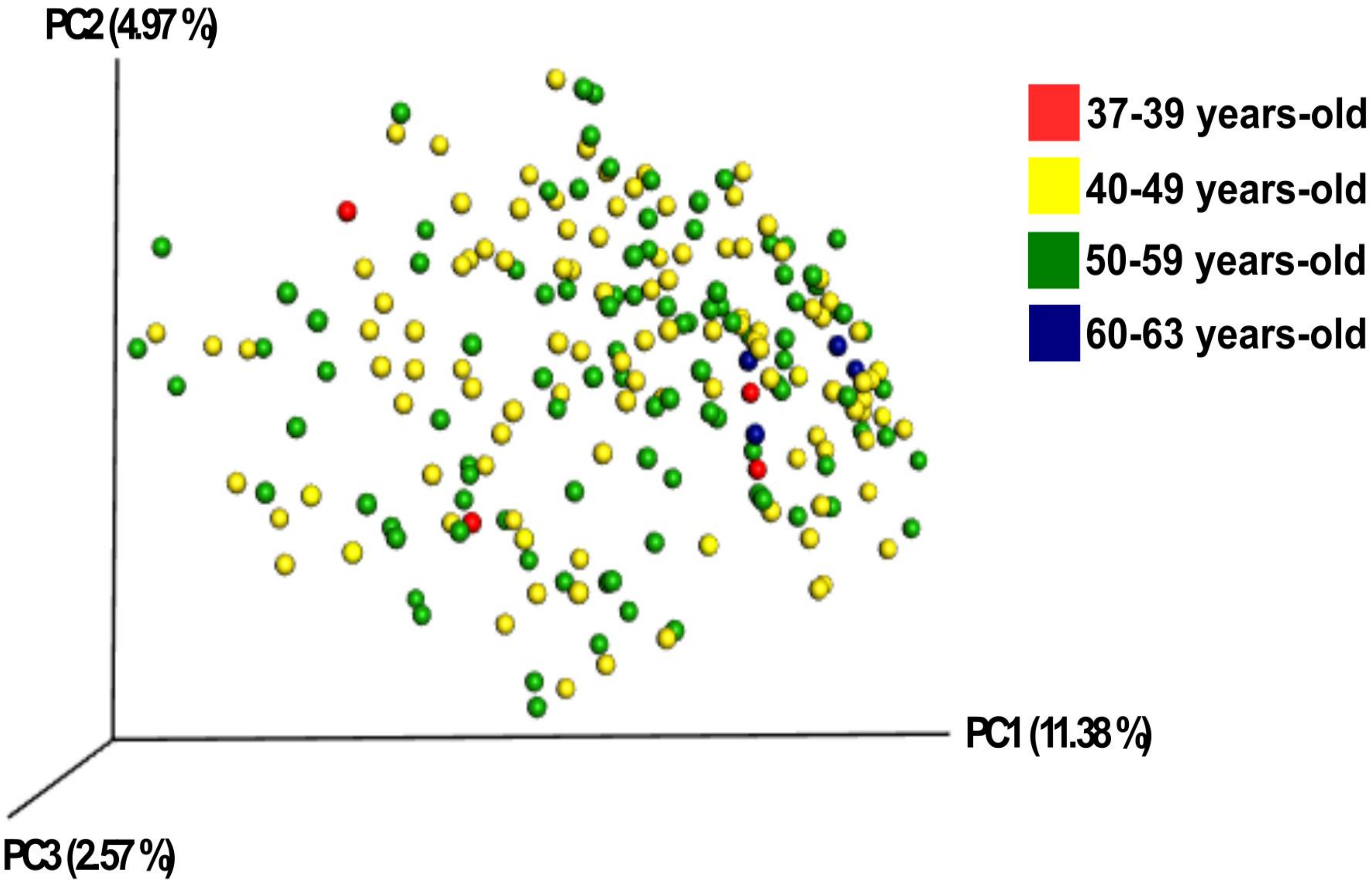

Figure S3 Bacterial beta diversity based on age. The figure shows a three-dimensional scatter plot, generated using Unweighted UniFrac analyses, showing for each dot, the distance along each of the three principal coordinates (PCoA) axis. Each dot represents the microbial community for each individual of all groups stratified by age range: Red 37-39, Yellow 40-49, Green 50-59, and Blue 60-63.

Table S1. Comparisons among pairs of groups from general characteristics.

Group 1	Group 2	p-value	q-value
**Waist circumference (cm)**			
A) CO	C) T2D−P	0.001	<0.001
**Ha1c (%)**			
A) CO	B) PRE	<0.001	<0.001
A) CO	C) T2D−No−M	<0.001	<0.001
A) CO	D) T2D−M	<0.001	<0.001
A) CO	E) T2D−P	<0.001	<0.001
A) CO	F) T2D−P+I	<0.001	<0.001
B) PRE	C) T2D−No−M	<0.001	<0.015
B) PRE	F) T2D−P+I	<0.001	<0.001
**Fasting Glucose (mg/dl)**			
A) CO	B) PRE	<0.001	<0.001
A) CO	C) T2D−No−M	<0.001	<0.001
A) CO	D) T2D−M	<0.001	<0.001
A) CO	E) T2D−P	<0.001	<0.001
A) CO	F) T2D−P+I	<0.001	<0.001
B) PRE	E) T2D−P	<0.001	<0.010
B) PRE	F) T2D−P+I	<0.001	<0.001
**Kcal (kcal/day)**			
A) CO	F) T2D−P+I	<0.001	<0.001
A) CO	E) T2D−P	<0.001	0.022
B) PRE	F) T2D−P+I	<0.002	0.039
**Protein (g/day)**			
A) CO	E) T2D−P	<0.001	0.011

A) CO	F) T2D−P+I	<0.001	0.002
**Lipids (g/day)**			
A) CO	F) T2D−P+I	<0.001	0.001
A) CO	E) T2D−P	0.002	0.043
B) PRE	F) T2D−P+I	<0.001	0.007
**Carbohydrates (g/day)**			
A) CO	F) T2D−P+I	0.001	<0.001
**Cholesterol (mg/dl)**			
C) T2D−No−M	E) T2D−P	0.001	<0.001
C) T2D−No−M	F) T2D−P+I	<0.001	0.002
B) PRE	E) T2D−P	<0.001	0.021
**Triglycerides (mg/dl)**			
A) CO	F) T2D−P+I	0.002	0.047
**HDL (mg/dl)**			
A) CO	E) T2D−P	0.002	0.036
B) PRE	E) T2D−P	0.002	0.037

P-value was calculated according to Kruskal Wallis test and Bonferroni correction such a q-value for non-parametric data and ANOVA for parametric with Tukey such as q-value thought Post-Hoc test. Control, CO; Prediabetes, PRE; T2D no medication, T2D−No−M; T2D Metformin, T2D−M; T2D polypharmacy, T2D−P; T2D polypharmacy + insulin, T2D−P+I. Polypharmacy - hypoglycemic agents and other drugs such as antihypertensive, lipid-lowering among others.

Table S2. Mean of relative abundances (%) between groups at phylum level.

**Phylum**	**A) CO**	**B) PRE**	***p*-value**	**FDR**	**Bonferroni**
Firmicutes	61.95	56.07	0.19	0.90	1
Bacteroidetes	33.21	40.41	0.13	0.88	1
Proteobacteria	3.34	2.42	0.45	0.97	1
Actinobacteria	1.16	0.82	0.56	0.98	1
	**A) CO**	**C) T2D−No−M**	**P**	**FDR**	**Bonferroni**
Firmicutes	61.95	43.11	0.01	0.18	0.21
Bacteroidetes	33.21	53.18	0.01	0.18	0.36
Proteobacteria	3.34	2.79	0.78	1	1
Actinobacteria	1.16	0.76	0.57	1	1
	**C) T2D−No−M**	**B) PRE**	**P**	**FDR**	**Bonferroni**
Firmicutes	43.11	56.07	0.09	0.90	1
Bacteroidetes	53.18	40.41	0.12	0.90	1
Proteobacteria	2.79	2.42	0.86	1	1
Actinobacteria	0.76	0.82	0.91	1	1
	**A) CO**	**D) T2D−M**	**P**	**FDR**	**Bonferroni**
Firmicutes	61.95	31.98	<0.01 ǂǂ	0.01	0.03 *****
Bacteroidetes	33.21	64.74	<0.01 ǂǂ	0.01	0.03 *****
Proteobacteria	3.34	2.65	0.73	1	1
Actinobacteria	1.16	0.51	0.24	0.86	1
	**C) T2D−No−M**	**D) T2D−M**	**P**	**FDR**	**Bonferroni**
Firmicutes	43.11	31.98	0.20	1	1
Bacteroidetes	53.18	64.74	0.19	1	1
Proteobacteria	2.79	2.65	0.92	1	1
Actinobacteria	0.76	0.51	0.50	1	1
	**E) T2D−P**	**F) T2D−P+I**	**P**	**FDR**	**Bonferroni**

Firmicutes	66.83	62.35	0.46	1	1
Bacteroidetes	29.00	33.58	0.47	1	1
Proteobacteria	2.66	2.38	0.83	1	1
Actinobacteria	1.30	1.01	0.50	1	1
	**D) T2D−M**	**F) T2D−P+I**	**P**	**FDR**	**Bonferroni**
Firmicutes	31.98	62.35	<0.01 †	0.03	0.03 *
Bacteroidetes	64.74	33.58	<0.01 †	0.03	0.06
Proteobacteria	2.65	2.38	0.82	1	1
Actinobacteria	0.51	1.01	0.07	0.55	1
	**C) T2D−No−M**	**F) T2D−P+I**	**P**	**FDR**	**Bonferroni**
Firmicutes	43.11	62.35	0.00	0.15	0.15
Bacteroidetes	53.18	33.58	0.01	0.22	0.45
Proteobacteria	2.79	2.38	0.73	1	1
Actinobacteria	0.76	1.01	0.42	1	1
	**C) T2D−No−M**	**E) T2D−P**	**P**	**FDR**	**Bonferroni**
Firmicutes	43.11	66.83	0.01	0.12	0.18
Bacteroidetes	53.18	29.00	0.01	0.12	0.24
Proteobacteria	2.79	2.66	0.94	1	1
Actinobacteria	0.76	1.30	0.40	0.86	1
	**E) T2D−P**	**D) T2D−M**	**P**	**FDR**	**Bonferroni**
Firmicutes	66.83	31.98	<0.01 ††	0.01	0.03 *
Bacteroidetes	29.00	64.74	<0.01 ††	0.01	0.03 *
Proteobacteria	2.66	2.65	1.00	1	1
Actinobacteria	1.30	0.51	0.19	0.99	1
	**A) CO**	**E) T2D−P**	**P**	**FDR**	**Bonferroni**
Firmicutes	61.95	66.83	0.43	1	1
Bacteroidetes	33.21	29.00	0.48	1	1

Proteobacteria	3.34	2.66	0.70	1	1
Actinobacteria	1.16	1.30	0.86	1	1
	**A) CO**	**F) T2D−P+I**	**P**	**FDR**	**Bonferroni**
Firmicutes	61.95	62.35	0.95	1	1
Bacteroidetes	33.21	33.58	0.94	1	1
Proteobacteria	3.34	2.38	0.44	1	1
Actinobacteria	1.16	1.01	0.87	1	1
	**D) T2D−M**	**B) PRE**	**P**	**FDR**	**Bonferroni**
Firmicutes	31.98	56.07	<0.01 ɫ	0.04	0.09
Bacteroidetes	64.74	40.41	<0.01 ɫ	0.04	0.09
Proteobacteria	2.65	2.42	0.91	1	1
Actinobacteria	0.51	0.82	0.48	1	1
	**B) PRE**	**E) T2D−P**	**P**	**FDR**	**Bonferroni**
Firmicutes	56.07	66.83	0.09	0.71	1
Bacteroidetes	40.41	29.00	0.09	0.71	1
Proteobacteria	2.42	2.66	0.88	1	1
Actinobacteria	0.82	1.30	0.27	1	1
	**B) PRE**	**F) T2D−P+I**	**P**	**FDR**	**Bonferroni**
Firmicutes	56.07	62.35	0.22	1	1
Bacteroidetes	40.41	33.58	0.21	1	1
Proteobacteria	2.42	2.38	0.97	1	1
Actinobacteria	0.82	1.01	0.55	1	1

T-test non parametric was measure between the mean of each group, FDR and Bonferroni correction after that, *p-value* to accept the hypothesis was <0.05. FDR - False Discovery Rates. ***** means a statistical difference between the groups with Bonferroni *p-values*. Control, CO; Prediabetes, PRE; T2D no medication, T2D−No−M; T2D Metformin, T2D−M; T2D polypharmacy, T2D−P; T2D polypharmacy + insulin, T2D−P+I. ɫ = *p*-value <0.01, ɫɫ = p-value <0.001.

Table S3. Comparisons between groups through alpha diversity indexes.

Group 1	Group 2	Group 1 mean	Group 1 std	Group 2 mean	Group 2 std	*p-value*
**Observed**						
E) T2D−P	D) T2D−M	149.68	28.14	119.32	42.68	0.06
C) T2D−No−M	E) T2D−P	135.36	34.83	149.68	28.14	0.34
A) CO	E) T2D−P	168.86	47.16	149.68	28.14	0.14
B) PRE	F) T2D−P+I	163.23	49.23	159.44	33.29	0.68
F) T2D−P+I	E) T2D−P	159.44	33.29	149.68	28.14	0.36
B) PRE	A) CO	163.23	49.23	168.86	47.16	0.56
C) T2D−No−M	D) T2D−M	135.36	34.83	119.32	42.68	0.35
A) CO	D) T2D−M	168.86	47.16	119.32	42.68	0.02*
C) T2D−No−M	A) CO	135.36	34.83	168.86	47.16	0.07
F) T2D−P+I	D) T2D−M	159.44	33.29	119.32	42.68	0.03*
B) PRE	E) T2D−P	163.23	49.23	149.68	28.14	0.38
B) PRE	D) T2D−M	163.23	49.23	119.32	42.68	0.02*
C) T2D−No−M	B) PRE	135.36	34.83	163.23	49.23	0.14
F) T2D−P+I	A) CO	159.44	33.29	168.86	47.16	0.37
C) T2D−No−M	F) T2D−P+I	135.36	34.83	159.44	33.29	0.10
**Chao1**						
Group 1	Group 2	Group 1 mean	Group 1 std	Group 2 mean	Group 2 std	*p-value*
E) T2D−P	D) T2D−M	311.94	65.96	234.37	90.72	0.02*
C) T2D−No−M	E) T2D−P	256.40	62.40	311.94	65.96	0.04*
A) CO	E) T2D−P	348.33	111.18	311.94	65.96	0.28
B) PRE	F) T2D−P+I	333.09	108.89	327.85	72.00	0.80
F) T2D−P+I	E) T2D−P	327.85	72.00	311.94	65.96	0.54

B) PRE	A) CO	333.09	108.89	348.33	111.18	0.52
C) T2D−No−M	D) T2D−M	256.40	62.40	234.37	90.72	0.52
A) CO	D) T2D−M	348.33	111.18	234.37	90.72	0.02*
C) T2D−No−M	A) CO	256.40	62.40	348.33	111.18	0.02*
F) T2D−P+I	D) T2D−M	327.85	72.00	234.37	90.72	0.02*
B) PRE	E) T2D−P	333.09	108.89	311.94	65.96	0.55
B) PRE	D) T2D−M	333.09	108.89	234.37	90.72	0.02*
C) T2D−No−M	B) PRE	256.40	62.40	333.09	108.89	0.03*
F) T2D−P+I	A) CO	327.85	72.00	348.33	111.18	0.47
C) T2D−No−M	F) T2D−P+I	256.40	62.40	327.85	72.00	0.01*

Shannon

Group 1	Group 2	Group 1 mean	Group 1 std	Group 2 mean	Group 2 std	*p-value*
E) T2D−P	D) T2D−M	5.22	0.76	4.65	0.90	0.21
C) T2D−No−M	E) T2D−P	5.07	0.74	5.22	0.76	0.71
A) CO	E) T2D−P	5.53	0.87	5.22	0.76	0.29
B) PRE	F) T2D−P+I	5.46	0.89	5.48	0.65	0.92
F) T2D−P+I	E) T2D−P	5.48	0.65	5.22	0.76	0.32
B) PRE	A) CO	5.46	0.89	5.53	0.87	0.79
C) T2D−No−M	D) T2D−M	5.07	0.74	4.65	0.90	0.31
A) CO	D) T2D−M	5.53	0.87	4.65	0.90	0.02*
C) T2D−No−M	A) CO	5.07	0.74	5.53	0.87	0.18
F) T2D−P+I	D) T2D−M	5.48	0.65	4.65	0.90	0.02*
B) PRE	E) T2D−P	5.46	0.89	5.22	0.76	0.34
B) PRE	D) T2D−M	5.46	0.89	4.65	0.90	0.03*
C) T2D−No−M	B) PRE	5.07	0.74	5.46	0.89	0.26
F) T2D−P+I	A) CO	5.48	0.65	5.53	0.87	0.81

C) T2D−No−M	F) T2D−P+I	5.07	0.74	5.48	0.65	0.22
**Simpson**						
Group 1	Group 2	Group 1 mean	Group 1 std	Group 2 mean	Group 2 std	*p-value*
E) T2D−P	D) T2D−M	0.92	0.08	0.89	0.06	0.72
C) T2D−No−M	E) T2D−P	0.92	0.04	0.92	0.08	0.80
A) CO	E) T2D−P	0.93	0.06	0.92	0.08	0.60
B) PRE	F) T2D−P+I	0.93	0.06	0.94	0.04	0.68
F) T2D−P+I	E) T2D−P	0.94	0.04	0.92	0.08	0.41
B) PRE	A) CO	0.93	0.06	0.93	0.06	0.89
C) T2D−No−M	D) T2D−M	0.92	0.04	0.89	0.06	0.32
A) CO	D) T2D−M	0.93	0.06	0.89	0.06	0.20
C) T2D−No−M	A) CO	0.92	0.04	0.93	0.06	0.74
F) T2D−P+I	D) T2D−M	0.94	0.04	0.89	0.06	0.06
B) PRE	E) T2D−P	0.93	0.06	0.92	0.08	0.60
B) PRE	D) T2D−M	0.93	0.06	0.89	0.06	0.19
C) T2D−No−M	B) PRE	0.92	0.04	0.93	0.06	0.70
F) T2D−P+I	A) CO	0.94	0.04	0.93	0.06	0.65
C) T2D−No−M	F) T2D−P+I	0.92	0.04	0.94	0.04	0.37

The results appear like mean and ± standard deviation - std. *P-value* was calculated to compares alpha diversities based on a two-sample t-test using a non-parametric method and the default number of Monte Carlo permutations in order to find different significances among groups. Control, CO; Prediabetes, PRE; T2D−No−Medication, T2D−No−M; T2D−Metformin, T2D−M; T2D−Polypharmacy, T2D−P; T2D−Polypharmacy + insulin, T2D−P+I. Polypharmacy - hypoglycemic agents and other drugs such as antihypertensive, lipid-lowering among others. *, means significant differences between pairs of groups.

Table S4. Comparisons among groups through beta diversity with ANOSIM.

Group1	Group2	Sample size	Groups	Test statistic	*p-value*
All	NA	217	6	0.10	0.01
A) CO	C) T2D−No−M	90	2	0.26	0.01
A) CO	D) T2D−M	90	2	0.30	0.01
A) CO	E) T2D−P	98	2	0.08	0.13
A) CO	F) T2D−P+I	113	2	0.01	0.38
A) CO	B) PRE	130	2	0.01	0.39
D) T2D−M	C) T2D−No−M	28	2	0.02	0.23
C) T2D−No−M	B) PRE	68	2	0.20	0.04
E) T2D−P	C) T2D−No−M	36	2	0.19	0.01
E) T2D−P	D) T2D−M	36	2	0.17	0.02
F) T2D−P+I	D) T2D−M	51	2	0.31	0.01
F) T2D−P+I	C) T2D−No−M	51	2	0.35	0.01
F) T2D−P+I	E) T2D−P	59	2	0.05	0.13
B) PRE	D) T2D−M	68	2	0.24	0.01
B) PRE	E) T2D−P	76	2	0.07	0.17
B) PRE	F) T2D−P+I	91	2	0.01	0.37

P-value was calculated to compares beta diversities using a distance matrix as the primary input and mapping file. ANOSIM method such a non-parametric test and the default number of permutations in order to find different significances among groups. Control, CO; Prediabetes, PRE; T2D−No−Medication, T2D−No−M; T2D−Metformin, T2D−M; T2D−Polypharmacy, T2D−P; T2D−Polypharmacy + insulin, T2D−P+I. Polypharmacy - hypoglycemic agents and other drugs such as antihypertensive, lipid-lowering among others. NA – Non applicable.

Table S5. Enrich bacteria in the indicated sets according to LEfSe analyses after Benjamini-Hochberg correction.

Taxonomic Hierarchy	Enriched in GROUP	LDA	p-value	q-value
Bacteria|Proteobacteria|Betaproteobacteria	A) CO	2.90	<0.0001	0.0002
Bacteria|Proteobacteria|Betaproteobacteria|Burkholderiales	A) CO	2.90	<0.0001	0.0002
Bacteria|Proteobacteria|Betaproteobacteria|Burkholderiales|*Alcaligenaceae*	A) CO	2.89	<0.0001	0.0002
Bacteria|Proteobacteria|Betaproteobacteria|Burkholderiales|*Comamonadaceae*	B) PRE	2.07	0.0004	0.0072
Bacteria|Proteobacteria|Betaproteobacteria|Burkholderiales|*Alcaligenaceae*|*Sutterella*	C) T2D−No−M	2.85	<0.0001	0.0004
Bacteria|Proteobacteria|Betaproteobacteria|Burkholderiales|*Comamonadaceae*|*Pelomonas*	D) T2D−M	2.61	<0.0001	<0.0001
Bacteria|Bacteroidetes|Bacteroidia	D) T2D−M	4.43	0.0001	0.0013
Bacteria|Bacteroidetes|Bacteroidia|Bacteroidales	D) T2D−M	4.43	0.0001	0.0013
Bacteria|Acidobacteria|Acidobacteriia|Acidobacteriales|*Koribacteraceae*	D) T2D−M	2.78	0.0013	0.0243
Bacteria|Acidobacteria|Acidobacteriia	D) T2D−M	2.58	0.0013	0.0243
Bacteria|Acidobacteria|Acidobacteriia|Acidobacteriales	D) T2D−M	2.74	0.0013	0.0243
Bacteria|Firmicutes|Clostridia|Clostridiales|*Ruminococcaceae*|*Oscillospira*	E) T2D−P and T2D−P+I	3.03	0.0001	0.0018
Bacteria|Firmicutes|Clostridia|Clostridiales|*Lachnospiraceae*|*Roseburia*	E) T2D−P and T2D−P+I	2.94	0.0001	0.0022

The p-value was calculated using Linear Discriminant Analysis Effect Size (LEfSe) analysis, and the q-value was calculated using the Benjamini-Hochberg test. Control, CO; Prediabetes, PRE; T2D no medication, T2D−No−M; T2D Metformin, T2D−M; T2D polypharmacy, T2D−P; T2D polypharmacy + insulin, T2D−P+I. Polypharmacy - hypoglycemic agents and other drugs such as antihypertensive, lipid-lowering among others. For CO, the relative abundance of the family *Alcaligenaceae* was 0.1934%, order Burkholderiales 0.0007%, and class Betaproteobacteria 0.00001%; for PRE the relative abundance of the family *Comamonadaceae* was 0.0747%; for T2D−No−M, the relative abundance of genus *Sutterella* was 0.9734%; for T2D−M, the relative abundance of the class Acidobacteriia, order Acidobacteriales, family *Koribacteraceae* was 0.0003%, for genus *Pelomonas* was 0.0004%, for the class Bacteroidia, order Bacteroidales was 0.4185%; finally for T2D−P and T2D−P+I, the abundance was genus *Roseburia* 2.89% and genus *Oscillospira*, 3.24%.

Table S6. Select microbial taxa significantly linked to clinical parameters, anthropometrical profile and dietary intake.

**Taxonomic Hierarchy**	**Variable**	***p*-value**	***q*-value**
n=217			
Bacteria|Firmicutes|Bacilli|Lactobacillales|*Enterococcaceae*	Age	0.0001	0.0870
Bacteria|Bacteroidetes|Bacteroidia|Bacteroidales|*Paraprevotellaceae*|*Prevotella*	Gender	0.0002	0.0870
Bacteria|Proteobacteria|Alphaproteobacteria|Sphingomonadales|*Sphingomonadaceae*|*Kaistobacter*	Lipid intake	0.0001	0.0870
Bacteria|Proteobacteria|Gammaproteobacteria|Enterobacteriales|*Enterobacteriaceae*|*Erwinia*	Systolic BP	0.0005	0.1692
n=99			
Bacteria|Firmicutes|Clostridia|Clostridiales|*Lachnospiraceae*|*Dorea*	Physical activity	0.0001	0.2093
Bacteria|Firmicutes|Bacilli|Lactobacillales|*Enterococcaceae*	Body fat (%)	0.0004	0.2093
Bacteria|Firmicutes|Bacilli|Lactobacillales|*Enterococcaceae*	Body fat (Kg)	0.0003	0.2093
Bacteria|Fusobacteria|Fusobacteriia|Fusobacteriales|*Fusobacteriaceae*|*Fusobacterium*	Weight	0.0004	0.2093

The *p*-value was calculated using Multivariate Association with Linear Models (MaAsLin) algorithm, and the *q*-value was calculated using FDR to corrected *p*-value. Systolic BP – Systolic Blood Pressure.

Table S7. Gene content prediction between prediabetes and type two diabetes with metformin groups, after FDR correction.

Metabolic Pathway	B) PRE (%)	D) T2D−M (%)	*p*-value	*q*-value
Energy metabolism	0.944	*0.998	0.006	0.040
Carbohydrate metabolism				
Amino sugar and nucleotide sugar metabolism	1.481	*1.617	0.002	0.031
Propanoate metabolism	*0.494	0.446	0.007	0.042
Amino acid metabolism				
Glycine, serine and threonine metabolism	0.833	*0.890	0.003	0.033
Lipid metabolism				
Sphingolipid metabolism	0.238	*0.313	0.005	0.037
Glycan biosynthesis and metabolism				
Lipopolysaccharide biosynthesis	0.229	*0.400	0.007	0.042
Xenobiotics biodegradation and metabolism				
Benzoate degradation	*0.212	0.167	0.008	0.046
Atrazine degradation	*0.013	0.008	0.002	0.029
Dioxin degradation	*0.053	0.036	0.003	0.032
Xylene degradation	*0.051	0.035	0.004	0.033
Styrene degradation	*0.019	0.013	0.003	0.031

The % is reported as the mean of the relative frequencies ± standard deviation. The *p*-value was calculated using Welch's t-test, and the *q*-value was calculated using Benjamini-Hochberg such as FDR test. Prediabetes, PRE; T2D Metformin, T2D−M. An asterisk (*) indicates the highest value among groups. See Figure 7.

© 2019 by the authors. Submitted for possible open access publication under the terms and conditions of the Creative Commons Attribution (CC BY) license (http://creativecommons.org/licenses/by/4.0/).
